# Inequities in skilled attendance at birth in Namibia: A decomposition analysis

**DOI:** 10.1186/1471-2393-11-34

**Published:** 2011-05-14

**Authors:** Eyob Zere, Doyin Oluwole, Joses M Kirigia, Chris N Mwikisa, Thomas Mbeeli

**Affiliations:** 1 Africa's Health in 2010, Academy for Educational Development, 1825 Connecticut Avenue, Washington, DC 20009, USA; 2 World Health Organization, Regional Office for Africa, Brazzaville, Congo, Africa; 3 Ministry of Health and Social Services, Windhoek, Namibia, Africa

## Abstract

**Background:**

The fifth Millennium Development Goal (MDG5) aims at improving maternal health. Globally, the maternal mortality ratio (MMR) declined from 400 to 260 per 100000 live births between 1990 and 2008. During the same period, MMR in sub-Saharan Africa decreased from 870 to 640. The decreased in MMR has been attributed to increase in the proportion of deliveries attended by skilled health personnel. Global improvements maternal health and health service provision indicators mask inequalities both between and within countries. In Namibia, there are significant inequities in births attended by skilled providers that favour those that are economically better off. The objective of this study was to identify the drivers of wealth-related inequalities in child delivery by skilled health providers.

**Methods:**

Namibia Demographic and Health Survey data of 2006-07 are analysed for the causes of inequities in skilled birth attendance using a decomposable health concentration index and the framework of the Commission on Social Determinants of Health.

**Results:**

About 80.3% of the deliveries were attended by skilled health providers. Skilled birth attendance in the richest quintile is about 70% more than that of the poorest quintile. The rate of skilled attendance among educated women is almost twice that of women with no education. Furthermore, women in urban areas access the services of trained birth attendant 30% more than those in rural areas. Use of skilled birth attendants is over 90% in Erongo, Hardap, Karas and Khomas Regions, while the lowest (about 60-70%) is seen in Kavango, Kunene and Ohangwena. The concentration curve and concentration index show statistically significant wealth-related inequalities in delivery by skilled providers that are to the advantage of women from economically better off households (C = 0.0979; P < 0.001).

Delivery by skilled health provider by various maternal and household characteristics was 21 percentage points higher in urban than rural areas; 39 percentage points higher among those in richest wealth quintile than the poorest; 47 percentage points higher among mothers with higher level of education than those with no education; 5 percentage points higher among female headed households than those headed by men; 20 percentage points higher among people with health insurance cover than those without; and 31 percentage points higher in Karas region than Kavango region.

**Conclusion:**

Inequalities in wealth and education of the mother are seen to be the main drivers of inequities in the percentage of births attended by skilled health personnel. This clearly implies that addressing inequalities in access to child delivery services should not be confined to the health system and that a concerted multi-sectoral action is needed in line with the principles of the Primary health Care.

## Background

There is increasing evidence on the existence of pervasive inequalities in health and health care that are related to socio-economic position as may be measured by household income/expenditure/wealth, occupation, gender, area of residence etc. both between and within countries [1,2]. The existing evidence unequivocally reveals that morbidity and mortality are more prevalent at the lower end of the socio-economic ladder. In contrast, access to health services is concentrated among those at the upper end of the socio-economic spectrum.

The overwhelming evidence on socio-economic inequalities in health and health care has led to renewed interest globally and nationally to understand the causes of health sector inequalities and develop appropriate policy responses [3].

The differential in maternal health indicators is perhaps the largest differential in health status between rich and poor countries [4,5]. In Sub-Saharan Africa, the adjusted maternal mortality ratio in 2008 was 640 per 100,000 live births, as compared to 14 per 100,000 in the developed regions. Similarly, while the life time risk of maternal death is 1 in 31 in Sub-Saharan Africa, the corresponding figure in the developed regions of the world is 1 in 4,300 [6].

In the period 2000-2008, about 96% of child deliveries were attended by skilled health personnel in the European Region of the World Health Organization. However, despite global improvement in the proportion of women delivered by skilled health workers, the situation in Africa has not changed [7]. In the African Region only 47% of births were attended by skilled health personnel. The situation in Namibia is much better compared to this average - about 81% of births attended by skilled health personnel.

Data from low and middle-income countries also show significant within country gradients in health outcomes and utilization of health services. In the poorest 20% of the population, an infant is more than twice as likely to die before the age of 1 year and an under-five child is more than three times as likely to be stunted (short-for-age) compared to children from the 20% economically better-off households [8].

At the dawn of the current Millennium, World Leaders agreed to reduce maternal mortality by 75% in 2015 compared to its 1990 levels. One of the indicators for MDG 5 is the proportion of deliveries attended by trained health providers, which include doctors, nurses or midwives. Delivery by trained providers is necessary to reduce maternal mortality and is easy to monitor regularly and at reasonably short intervals compared to maternal mortality.

Global improvements mask inequalities both between and within countries. In Namibia, there are significant inequities in births attended by skilled providers that favour those that are economically better off [9]. Identifying the determinants of inequities in health and health care is essential to designing equitable interventions [10].

In line with the conceptual framework of the WHO Commission on Social Determinants of Health, access to delivery services by skilled health providers is shaped by political, social and economic forces. It then follows that addressing inequities requires a concerted multi-sectoral action, which is also in line with the Principles of Primary Health Care. The objective of this study was to identify the drivers of wealth-related inequalities in child delivery by skilled health providers using a decomposable health concentration index and the framework of the Commission on Social Determinants of Health (CSDH).

## Brief country profile

Namibia is a country in the South Western part of Africa covering a land area of 824,000 square kilometers. According to the 2001 population and housing census, the population was about 1.8 million with an inter-censal growth rate of 2.6% per annum [11]. The country is divided into 13 Administrative Regions, which also correspond to the health regions.

Namibia is an upper middle income country [12] and one of those with the highest income inequality in the world [13]. Table 1 below depicts data on selected health and development indicators [13-16].

**Table 1:** Namibia: selected health and development indicators

Indicator	Value
GDP per capita, 2008 (US$)	4,149
Income Gini index, 2000-2010	74.3
Human Development Index (HDI), 2010	0.606
Inequality-adjusted HDI, 2010	0.338
HDI rank out of 182 countries	108
GNP per capita rank minus HDI rank, 2010	-14
Life expectancy at birth, 2007 (years)	59
Infant mortality rate per 1000 live births, 2002-2006	46
Under-five mortality rate per 1000 live births, 2002-2006	69
Total fertility rate, 2006-2007	3.6
Maternal mortality ratio per 100,000 live births, 1998-2007	449
Percentage of pregnant women receiving antenatal care from a skilled provider, 2006-2007	94.6
Percentage of deliveries by a skilled provider, 2006-2007	81.4
Adult (15-49 years) HIV prevalence rate, 2008 (%)	15.3
Health expenditure per capita (real 2006 US$)	276
Physicians per 10,000 population 2000-2007	3
Nursing and midwifery personnel per 10,000 population, 2000-2007	31
Hospital beds per 10,000 population, 2000-2008	33
Antenatal care from skilled provider - at least one visit, 2006/2007	94.6
Antenatal care from skilled provider - 4+ visits	70.4

As can be seen from Table 1, although the country is better off than many countries in sub-Saharan Africa in terms of resources for health and development, there is a significant amount of inequity and inefficiency. The potential loss in human development due to inequalities in each of the dimensions of the HDI (life expectancy at birth, gross national income per capita and schooling) amounts to about 44%. The HDI falls to a level which is less than that of countries classified as low human development (HDI = 0.393) [13]. Furthermore, the GNP per capita rank minus the HDI rank was -14 indicating inefficiency in translating resources into welfare, i.e. the country did not achieve the level of human development that could potentially have been achieved given its resources.

Antenatal care from a skilled provider (at least one visit) was about 95%. However, the proportion of pregnant women who received four or more antenatal care visits was only about 70%. The maternal mortality ratio of 449 per 100,000 live births in 2006/07 is a significant increase from the 1992 level, which was 225 per 100,000 live births. Apart from the direct and indirect causes of maternal mortality; limited access to emergency obstetric care and lack of transport and communication facilities also contribute to maternal mortality.

To reverse the increasing trend of maternal mortality and consequently accelerate the progress towards the achievement of the 5^th ^Millennium Development Goal (MDG 5) of reducing the maternal mortality ratio by three-quarters between 1990 and 2015, the Government has embarked on a number of initiatives, including development and implementation of a Roadmap for Accelerating the Reduction of Maternal and Neonatal morbidity and mortality.

## Methods

### Measuring inequalities

In measuring equity in a health outcome or access to health interventions, the following are required:

- indicator of the health intervention of interest (delivery by skilled health providers)

- a variable (stratifier) capturing socio-economic status against which the distribution is to be assessed (wealth); and

- a measure of socio-economic inequality to quantify the degree of inequity in the indicator variable of interest.

A concentration index (C) is used to measure wealth-related inequalities in the observed use of delivery services by skilled health providers. The concentration index of a health care variable *y *(utilization of delivery services by trained health providers) can be defined using the concentration curve that links the cumulative proportion of individuals ranked by wealth to the corresponding cumulative proportion of *y *(use of delivery services by trained health providers). The concentration curve plots shares of the health care variable (*y*) against quantiles of the measure of socio-economic status (asset-based wealth index) [17].

The concentration index is defined as twice the area between the concentration curve and the line of equality and assumes values between -1 and +1. A negative value of the concentration index denotes inequity in skilled care at birth that is to the advantage of the lower wealth quintiles implying that women of lower socio-economic status are delivered by skilled health providers more than their counterparts who are wealthier. In this case the concentration curve lies above the line of equality. On the other hand, a positive concentration index implies inequality in the use of delivery services by skilled providers that favours women who are wealthier (the concentration curve lies below the line of equality). When the value of the concentration index is zero, there are no wealth related inequalities in the use of delivery services by skilled providers. The concentration curve overlaps with the 45-degree line.

From individual level data, the concentration index can be computed using the following formula [18]:(1)

Where

*h_i _*is the health variable of interest (delivery by skilled health providers);

*μ *is the mean of *h_i_*;

*R_i _*is the fractional rank of individual *i *in the distribution of socio-economic position; and

(; *i *= 1 for the poorest and *i *= *n *for the richest).

### Decomposing the concentration index

Wagstaff *et al*. [19] demonstrated that the concentration index of a health variable is additively decomposable to the concentration indices of the determinants of that health variable. In other words, the concentration index of the health variable of interest can be expressed as the sum of the contributions of the various determinants of that variable, together with unexplained residual component.

In decomposing the concentration index of delivery by skilled providers, the following steps are pursued:

1. Regressing the health variable against its determinants:(2)

Where: *y_i _*= 1 if the delivery was conducted by a skilled health provider;

*x_k_*: a set of exogenous determinants of delivery by trained health provider;

*β_k_*: coefficient of determinant *x_k_*; and

*ε_i_*: random error term.

The dependent variable (delivery by skilled health personnel) is a binary variable with values of 1 (delivered by skilled provider) and 0 (otherwise). The linear probability model (LPM) in Equation 2 above has been used in order to satisfy the linearity assumption of the decomposition analysis, although the estimates are inefficient and the probability of delivery by skilled health providers may not fall within the conventional values of 0 ≤ *p *≤ 1 and has heteroskedastic errors [20]. However, the estimated probabilities from the LPM model have been constrained within the conventional values and a comparison with a probit model has not shown significant variations between the coefficients of the LPM and the marginal (or average) effects of the probit regression derived using the *dprobit *Stata command [17]. Furthermore, to adjust for heteroskedasticity, the predicted values from the regression model have been saved and used as weights to run weighted least squares (WLS) using the "*aweight*" option in Stata [21]

2. Calculating concentration indices for the health variable and for its determinants (and generalized concentration index of the error term):

For any linearly additive regression model of the health variable of interest (*y_i_*) such as Equation 2 above, the concentration index for *y*, can be written as:(3)

Where:

*C_y_*: concentration index of skilled care at birth (i.e. concentration index of *y_i_*);

: mean value of determinant *x_k_*;

*μ*: mean of the outcome variable *y_i _*- that is the mean of deliveries by skilled health providers

*c_k_*: concentration index of determinant *x_k_*

*GC_ε_*: residual component that captures wealth-related inequality in skilled care at birth that is not accounted for by systematic variation in determinants across wealth groups.

The term in parenthesis in Equation 3 above expresses the impact of each determinant on the probability of delivery by skilled health providers. In other words, it denotes the elasticity (*η_k_*) of the outcome variable (delivery by skilled health providers) with respect to the determinant *x_k _*evaluated at the mean value of *y_i _*(delivery by skilled health providers). The concentration index of delivery by skilled health providers is thus a weighted sum of the inequality in each of its determinants, with the weights equal to the elasticities of the determinants:(4)

### The social determinants of health framework

**Figure 1:**
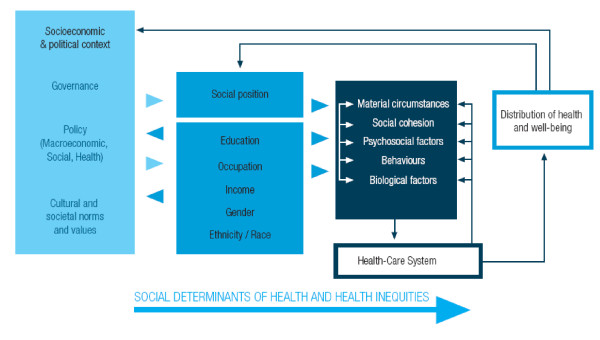
**Commission on Social Determinants of health conceptual framework**.

Report of the WHO Commission on the Social Determinants of Health revitalized the need for sustained and concerted efforts to achieve health equity through action on the social determinants of health. The Commission's social determinants framework takes a holistic view of inequities in health and health care within and between countries. Inequities in health/healthcare are caused by the unequal distribution of power, income, goods and services nationally and internationally (Figure 1) [22].

The social determinants of health are the circumstances in which people are born, grow up, live, work and age, and the systems put in place to deal with illness. These circumstances are in turn influenced by a wider set of forces: economics, social policies and politics [22].

The social determinants framework suggests that interventions to address health inequities have to be geared towards:

1. The circumstances of daily living, which include: differential exposure to health risks in early life, the social and physical environments and work associated with social stratification; and health care responses.

2. Structural drivers including the nature and degree of social stratification; biases, norms and values within society; global and national economic and social policy; and processes of governance at all levels.

As observed in Figure 1, the health system is an important social determinant of health influenced by and influencing the other social determinants. However, the health system is not the only social determinant of health. The effect of the each of the factors in Figure 1 in the genesis and perpetuation of health/health care inequities may vary from one country to another. It is therefore important to try to identify the effect of the various social determinants of health on health outcomes and access to health care in order to design evidence-based interventions and policy instruments.

### Data and Variables

Data from the Namibia Demographic and Health Survey 2006-07 was used for this study. The data is available on the MEASURE DHS website for registered users.

In the linear probability model of the determinants of delivery by skilled health providers and the decomposition analysis the following variables have been used:

1. Dependent variable: delivery by skilled health providers, which takes a value of 1 if the delivery has been attended by skilled health providers and a value of zero otherwise.

2. Independent variables:

• Region;

• Place of residence - urban/rural;

• Wealth as computed from the asset indices;

• Education of mother in years of schooling completed

• Head of household - a dummy where female household assumes a value of one; and

• Insurance coverage - a dummy with a value of one if the woman has insurance coverage.

In NDHS 2006-07, a representative two-stage probability sample of 10,000 households was selected. The first stage consisted of selection of 500 primary sampling units (PSUs) from a sampling frame of 3,750 PSUs with probability proportional to size; the size being the number of households in the 2001 Population Census. The second stage involved the systematic selection of 20 households in each PSU [14].

The demographic and health surveys do not contain data on household income or consumption expenditure. Instead wealth index is used as a proxy. The wealth index is based on household ownership of consumer goods (such as radio, television); dwelling characteristics; type of drinking water source; toilet facilities and other characteristics related to the household's socio-economic status. The asset indices are constructed using the method of principal component analysis (PCA) [14]. Studies have shown a close relationship between asset ownership and consumption expenditure in developing countries [23] and that household asset is a good indicator of the long-run economic status of households [24]

### Data analysis

Data was analyzed using STATA 10 statistical software and MS Excel.

## Results

### Descriptive statistics

About 80.3% of the deliveries were attended by skilled health providers. A breakdown by various maternal and household characteristics is provided in Table 2.

**Table 2:** Delivery by skilled health provider by various maternal and household characteristics

Characteristic	Delivery by skilled provider (%)
Place of residence:	
Rural	72.2
Urban	93.5
Wealth quintile:	
Poorest (Q1)	58.4
Poorer (Q2)	72.6
Middle (Q3)	84.8
Richer (Q4)	93.2
Richest (Q5)	97.2
Mother's level of education:	
No education	49.4
Primary	70.7
Secondary	91.5
Higher	96.2
Sex of household head:	
Female	82.7
Male	78.1
Health insurance coverage:	
No	78.0
Yes	97.5
Region:	
Caprivi	83.5
Erongo	90.6
Hardap	91.2
Karas	94.1
Kavango	63.1
Khomas	90.6
Kunene	63.6
Ohangwena	71.9
Omaheke	79.2
Omusati	88.2
Oshana	89.0
Oshikoto	79.3
Otjozondjupa	77.8

Delivery by skilled health providers is observed to differ by the various characteristics provided in Table 2. Most pronounced differences are seen by the household wealth status and mother's level of education. Skilled birth attendance in the richest quintile is about 70% more than that of the poorest quintile. The rate of skilled attendance among educated women is almost twice that of women with no education. Furthermore, women in urban areas access the services of trained birth attendant 30% more than those in rural areas.

Use of skilled birth attendants is over 90% in Erongo, Hardap, Karas and Khomas Regions, while the lowest (about 60-70%) is seen in Kavango, Kunene and Ohangwena. Parturient women in Erongo, Hardap, Karas and Khomas utilize the services of skilled attendants by more than 40% compared to those in Kavango. In six out of the thirteen regions, use of skilled delivery is less than the national average of 81%.

**Figure 2:**
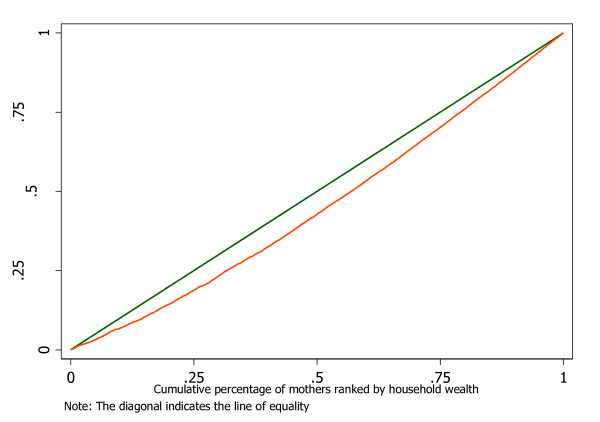
**Concentration curve: delivery by skilled health providers**.

The concentration curve (Figure 2) and concentration index show statistically significant wealth-related inequalities in delivery by skilled providers that are to the advantage of women from economically better off households (*C = 0.0979; P = < 0.001*). The concentration curve in Figure 2 lies below the main diagonal indicating that economically better off women are skilled birth attendants more than those who are economically worse off.

### Decomposition analysis

The decomposition analysis clarifies how each determinant of delivery by skilled providers contributes to the total wealth-related inequality in delivery by skilled health providers [25]. As can be observed from formula 4 (), the contribution of each determinant depends on: (i) its impact on delivery by skilled health providers (elasticity); and (ii) how unequally distributed over wealth the determinant is (its concentration index). The results of the decomposition analysis are depicted in Table 3.

**Table 3:** Results of the decomposition analysis

Variable	coefficient	Mean	Elasticity	C	Contribution to C	% contribution to C
Erongo	-0.0876227	0.0615	-0.00661	0.5441	-0.0036	-3.67
Hardap	0.0002425	0.0294	0.00001	0.3019	0.0000	0.00
Karas	-0.0033303	0.0292	0.00012	0.4219	-0.0001	-0.05
kavango	-0.1280143	0.1202	-0.01887	-0.4067	0.0077	7.84
Khomas	-0.0896233	0.1848	-0.02031	0.5900	-0.0120	-12.24
Kunene	-0.1518182	0.0378	-0.00703	-0.1337	0.0009	0.96
Ohangwena	-0.0607882	0.1143	-0.00852	-0.4373	0.0037	3.81
Omaheke	-0.0517934	0.0462	-0.00294	0.0567	-0.0002	-0.17
Omusati	0.0355298	0.0903	0.00394	-0.3560	-0.0014	-1.43
Oshana	0.0076805	0.0719	0.00068	0.0115	0.0000	0.01
Oshikoto	-0.0184259	0.0901	-0.00204	-0.2326	0.0005	0.48
Otjozondjupa	-0.0667059	0.0699	-0.00572	0.1933	-0.0011	-1.13
Urban residence	0.1046003	0.4154	0.05328	0.4802	0.0256	26.13
wealth	0.0000005	134746.8	0.07864	0.4205	0.0331	33.78
Education of mother	0.0291295	7.6238	0.27228	0.1540	0.0419	42.84
Female household head	0.0072470	0.4826	0.00429	-0.0561	-0.0002	-0.25
Has insurance coverage	-0.0075669	0.1418	-0.00132	0.5894	-0.0008	-0.79

***Residual (unexplained) *****0.0038**						

The concentration indices of Erongo, Hardap, Karas, Khomas have a high pro-wealthy concentration index (*P < 0.001*) implying that most of the relatively wealthy people inhabit this regions. In contrast, most of the relatively less wealthy people live in Ohangwena, Omusati and Oshikoto regions (*P < 0.001)*. The concentration indices for urban residence, education of mother, and insurance coverage have a statistically significant positive value indicating that they are more prevalent among the relatively wealthier segment of the population. On the other hand, female household heads are seen more among those that are less wealthy.

It is also observed that the three regions (Kavango, Kunene and Ohangwena) with the lowest rates of skilled birth attendance are mainly inhabited by the poor, as demonstrated by the negative concentration indices.

As discussed above, the contribution of each of the determinants to the total concentration index of delivery by skilled health providers depends on the impact of the determinant and its concentration index. A determinant that has a high impact but with no wealth-related gradient will have less contribution to the overall concentration index as opposed to one that with a high impact and high wealth-related inequality.

**Figure 3:**
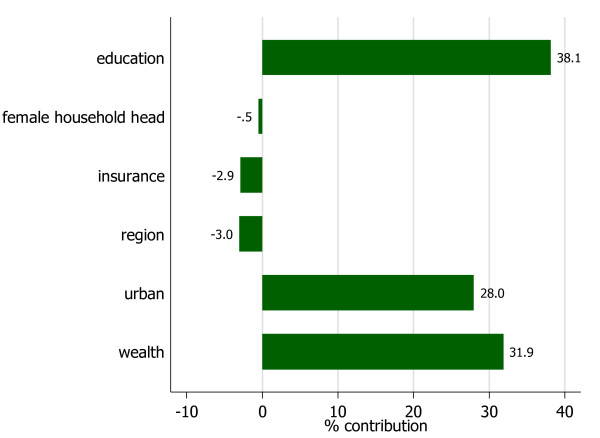
**Contribution of the determinants to the concentration index of delivery by skilled health providers**.

Figure 3 presents a summary of the contribution of the determinants to the overall concentration index of the variable, delivery by skilled health providers.

It is clearly observed that most of the inequality in delivery by skilled health providers that is to the advantage of the wealthier segment of the population is explained by inequalities in income, education and urban residence. The variables: region, insurance coverage and female-headed household seem to have inequality reducing effect, although not substantial.

## Discussion

To the best of our knowledge this is the first study of its kind in Namibia. An attempt is made to identify the drivers of wealth-related inequalities in child delivery by skilled health providers using a decomposable health concentration index and the framework of the Commission on Social Determinants of Health. Addressing inequities in health and health care requires action on the social determinants of health [1]. Design of appropriate evidence-based policy responses entails identification of those social determinants of health and health care that significantly influence inequities.

The findings of this study highlight the significant role that social determinants play in access to child delivery services by trained providers. Health systems are a social determinant of health that influence and are influenced by the other social determinants. Inequalities in wealth and education of the mother are seen to be the main drivers of inequities in the health system variable - delivery by trained health providers. This brings to the fore that addressing inequalities in access to child delivery services should not be confined to the health system and that a concerted multi-sectoral action is needed in line with the principles of the Primary Health Care [26].

In Namibia, inequality in wealth is found to be one of the major contributors of inequity in access to child delivery by trained providers. The country is one of those with the highest income inequality in the world. It is therefore imperative to address this unacceptably high level of income inequality, in order to improve inequities in access to delivery services by trained health providers. There has been a debate on the relationship between income inequality and health; findings and conclusions have been far from consistent [27]. However, income inequality is one of the markers of the unequal distribution of goods and services [22] including health-enhancing ones. Addressing inequities in delivery care by skilled attendants is essential for achieving MDG targets for maternal health [28]. Hence, addressing wealth inequalities contributes to improving equity in delivery by skilled attendants and consequently to achievement of the MDG related to improving maternal health. It is, however, important to note that redressing wealth inequalities alone can not be an effective intervention to inequities in access to maternity care in the absence of interventions that also tackle the other social determinants such as education [27].

The link between education of the mother and use of delivery services by trained health providers is well established [29]. Influences of maternal education can be observed in two ways: (i) education can improve the ability of individuals to produce health (without relying on health services) by influencing their life style; and (ii) increasing the use of health care services through improved knowledge, attitude and practice [30]. A study in Thailand using data from Multiple Indicator Cluster Survey (MICS) found that education of the mother is the major determinant of inequities in delivery by skilled health workers [31]. Our study indicates that inequality in maternal level of education is the major driver of inequities in delivery by trained health providers. Hence, bridging inequalities in the maternal levels of education is an important undertaking to narrow inequities in the use of delivery services by skilled health providers.

The other determinant of inequities in delivery by skilled providers is distribution according to residential location. It has been observed that there is a very high positive concentration index in urban residence implying that there is a high concentration of the economically better off segment of society in urban areas. It may be difficult in the current study to identify the mechanisms by which inequalities in urban residence influence inequities in delivery by skilled attendants. It may partly be explained by supply-side factors, where there is commonly a differential access to services favoring urban areas (urban-bias). There may also be a high concentration of the economically better off and the better educated in urban areas, which may contribute to the influence of inequalities of urban location to inequities in delivery by skilled attendants. It is thus important to carefully understand the mechanisms of influence and take appropriate equity-enhancing measures accordingly.

The social determinants of health such as education, income and place of residence are closely linked to access, experiences and benefits from health care, which is itself a social determinant of health [32]. The findings of this study are in line with this assertion of the WHO Commission on Social Determinants of Health. Therefore, addressing inequities in the use of delivery services by skilled health providers requires tackling these social determinants of health systems through a concerted multi-sectoral action. The four sets of Primary Health Care reforms that include: (i) the universal coverage reforms; (ii) service delivery reforms; (iii) public policy reforms; and (iv) leadership reforms [33] are very relevant here in bridging the observed inequities in the use of delivery services by skilled health providers and consequently contribute to the achievement of the MDG 5 targets.

## Conclusions

Most of the inequality in births attended by skilled health personnel, which is skewed towards the wealthier segment of the population, is explained by inequalities in income, education and urban residence. The region, insurance coverage and female-headed household variables seem to have inequality reducing effect, although not substantial.

Therefore, the fact that inequalities in wealth and education of the mother are the main drivers of inequities in the percentage of births attended by skilled health personnel implies that policy interventions for addressing inequalities in access to child delivery services should be multi-sectoral in line with the principles of the Primary health Care.

## List of abbreviations

C: Concentration index; CSDH: WHO Commission on Social Determinants of Health; DHS: Demographic and Health Survey; HDI: Human Development Index (HDI); LPM: Linear probability model; MDGs: Millennium Development Goals; MMR: Maternal mortality ratio; MICS: Multiple Indicator Cluster Survey; PHC: Primary health care

## Competing interests

The authors declare that they have no competing interests.

## Authors' contributions

EZ conceived the research, performed the analysis and drafted the manuscript. DO, TM, CNM and JMK contributed to the drafting of and review of the manuscript. All authors read and approved the final manuscript.

## Pre-publication history

The pre-publication history for this paper can be accessed here:

http://www.biomedcentral.com/1471-2393/11/34/prepub
